# Confounding Environmental Colour and Distribution Shape Leads to Underestimation of Population Extinction Risk

**DOI:** 10.1371/journal.pone.0055855

**Published:** 2013-02-11

**Authors:** Mike S. Fowler, Lasse Ruokolainen

**Affiliations:** 1 Population Ecology Group, Institut Mediterrani d’Estudis Avançats (UIB-CSIC), Esporles, Illes Balears, Spain; 2 Department of Biosciences, Swansea University, Swansea, United Kingdom; 3 Department of Biosciences, University of Helsinki, Helsinki, Finland; Albert Einstein College of Medicine, United States of America

## Abstract

The colour of environmental variability influences the size of population fluctuations when filtered through density dependent dynamics, driving extinction risk through dynamical resonance. Slow fluctuations (low frequencies) dominate in red environments, rapid fluctuations (high frequencies) in blue environments and white environments are purely random (no frequencies dominate). Two methods are commonly employed to generate the coloured spatial and/or temporal stochastic (environmental) series used in combination with population (dynamical feedback) models: autoregressive [AR(1)] and sinusoidal (1/*f*) models. We show that changing environmental colour from white to red with 1/*f* models, and from white to red or blue with AR(1) models, generates coloured environmental series that are not normally distributed at finite time-scales, potentially confounding comparison with normally distributed white noise models. Increasing variability of sample Skewness and Kurtosis and decreasing mean Kurtosis of these series alter the frequency distribution shape of the realised values of the coloured stochastic processes. These changes in distribution shape alter patterns in the probability of single and series of extreme conditions. We show that the reduced extinction risk for undercompensating (slow growing) populations in red environments previously predicted with traditional 1/*f* methods is an artefact of changes in the distribution shapes of the environmental series. This is demonstrated by comparison with coloured series controlled to be normally distributed using spectral mimicry. Changes in the distribution shape that arise using traditional methods lead to underestimation of extinction risk in normally distributed, red 1/*f* environments. AR(1) methods also underestimate extinction risks in traditionally generated red environments. This work synthesises previous results and provides further insight into the processes driving extinction risk in model populations. We must let the characteristics of known natural environmental covariates (e.g., colour and distribution shape) guide us in our choice of how to best model the impact of coloured environmental variation on population dynamics.

## Introduction

There is considerable interest in the importance of coloured stochastic processes (sometimes termed “noise”) across a wide range of scientific disciplines, from engineering and physics to evolutionary ecology and genetics [1]–[4]. An important characteristic of stochastic environmental variation is the rate at which changes in conditions occur over time or space. This can be characterized either as the serial correlation between observations (autocorrelation) or the dominant frequencies in the power spectrum of a fluctuating series. Both of these approaches aim to characterize the serial similarity in the data, which is often termed ‘colour’ in analogy with visible light: slowly changing series have a “red” spectrum where low frequencies dominate (positive autocorrelation), rapidly changing time series have a “blue” spectrum with high frequencies dominating (negative autocorrelation) and “white” series have an equal representation of all frequencies (zero autocorrelation). Natural sources of environmental variation tend to have a reddened spectrum, though there may be differences between the environments in aquatic and terrestrial ecosystems [5]–[7]. However, the estimation of colour from any time series is strongly time-scale dependent [3].

The colour of species’ responses to environmental variation has repeatedly been found to have an important impact on population extinction risk in simple, unstructured and more complex, structured dynamical models (reviewed in [3]). In populations with undercompensatory dynamics (those that return monotonically to equilibrium following a perturbation), counterintuitive results have been reported, where an initial increase in extinction risk with environmental reddening is followed by a decrease in very red, brown or black environments generated using sinusoidal (1/*f*) methods [8]. This trend differed from model predictions based on autoregressive [AR(1)] environmental series, which noted only an increase in extinction risk with environmental reddening under otherwise similar conditions [8]–[11]. This result is, however, sensitive to the parameter range explored and other model assumptions, e.g., minimum carrying capacity [12]. Environmental reddening is also expected to reduce the probability of extreme events in AR(1) models [12], which contradicts the observed increase in extinction risk. One explanation put forward for this discrepancy is an initial increase in the probability of a run of poor years in the environmental series [12]. This should, however, be compensated by an increase in the probability of runs of good years, reducing extinction risk. Therefore, we ask whether current insights are based on the effects of environmental colour, or other features of coloured environmental series?

Previous work has highlighted the importance of considering how the variance of a time-series changes with its colour at different time-scales [7], [11], [13], [14]. Here, we demonstrate that another simple, yet crucial feature of environmental time-series also changes with colour under different methods of generating coloured series: the shape of the frequency distribution for realised values of stochastic (environmental) time series changes with environmental reddening.

Most models assume that white (serially uncorrelated) noise is normally distributed (Gaussian), and comparison between different environmental colours is generally based on the implicit assumption that normality is retained as noise colour varies [10], [15]. While the distribution of the underlying stochastic component of the environmental series does not need to be normal [13], it is worth considering whether the distribution changes with colour and what impact this may have when interpreting results.

We examine the frequency distribution of AR(1) and 1/*f* coloured stochastic processes, comparing coloured series with normally distributed, white series. We show that coloured series tend to deviate from the normal distribution, which may have a confounding effect in studies using these methods. We then investigate population variability as a proxy for extinction risk in undercompensatory populations forced by environmental variation that either has a normal distribution for all colours, or where distribution shape varies with colour. We compare 1/*f* and AR(1) methods over a wider parameter space than previously studied for AR(1) noise [8], [9], [11], [12], to better understand when and why differences arise between these methods of generating coloured environmental series. We show that changes in the distribution shape of coloured environmental fluctuations lead to different extinction patterns under these alternative methods. These findings indicate that we need to add distribution shape to the list of important environmental characteristics, including mean, variance and colour, when evaluating the impact of environmental change on model and natural populations.

## Methods

### Stochastic Environmental Models and their Analysis

A simple, first-order autoregressive model was used to generate AR(1) coloured environmental time-series as follows:
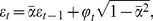
(1)where the value of the environmental variable (*ε*) is found over consecutive time steps (*t*) as a function of the desired temporal autocorrelation 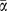 and a normally distributed random variable *φ* (with mean 0 and standard deviation 1). The square root term is usually included to maintain a constant variance (over infinite series) independently of 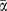. However, time series generated over finite time-scales with this method should be rescaled to give a desired variance over a specified scale [11], . We initially varied 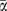 across 21 evenly spaced values between the limits [±0.999]. Sample autocorrelation coefficients, estimated from the realised series, are denoted *α* to distinguish them from the value of the coefficient used to generate the series, 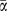.

Sinusoidal (1/*f*) environmental noise time-series were generated with the spectral synthesis method [8], [16], where amplitudes and periods of the desired spectral exponent (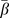; as above, sample spectral exponents estimated from the generated series are denoted *β*) were generated and sent to an inverse fast Fourier transform. In order to generate these time series (*ε_t_*), *n* random phases *θ_f_* were generated from a uniform distribution with limits [0, 2*π*], as well as *n* normally distributed random variables *ω_f_*, with 0 mean and unit variance. To generate the amplitudes for a desired spectral exponent (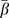) each normally distributed value (*ω_f_*) was multiplied by 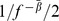 to form the amplitudes *af*. The complex coefficients (*CC*) were found as *CC* = *a_f_* exp(*iθ*), from which an inverse fast Fourier transform was taken. The real parts of this transform comprised the 1/*f* environmental noise time series (*ε_t_*). The desired spectral exponent defining time series colour, was initially varied across 21 evenly spaced values between [–2, 2], producing blue (negatively autocorrelated) and strongly reddened, or brown (positively autocorrelated) noise, respectively. This range of spectral exponents can also be approximately generated with AR(1) methods. We also examined alternative methods for generating 1/*f* signals, including those that generate spatio-temporally structured coloured series [9], [17], [18], but found no qualitative differences with the results presented below, when coloured series were appropriately controlled for independence.

We generated 1,000 replicate series of *T* = 10,000 steps for each parameter value for both AR(1) and 1/*f* series, then tested the normality of each series with a Jarque-Bera test. This compares the sample skewness and kurtosis statistics against the null hypothesis of those from series that are normally distributed (skewness = 0, kurtosis = 3). We recorded whether or not each series failed the normality test at significance level *a* = 0.05.

**Figure 1:**
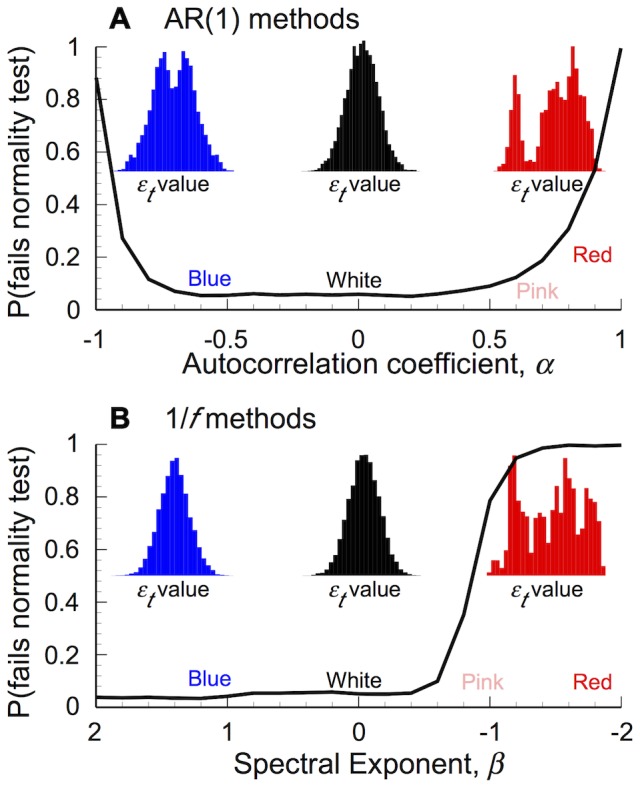
Coloured stochastic time-series are not normally distributed. The proportion of coloured stochastic (environmental) time-series that fail a normality test increases as they change colour, even when the same methods produce normally distributed white noise (10,000 step series; 1000 replicates for each parameter value), under both (A) Autoregressive and (B) Spectral synthesis methods (*x*-axis values reversed for comparison). Inlays illustrate frequency distributions for series of *ε_t_* values from sample blue (A: *α*≈**−**0.999, B: *β*≈1), white (*α*, *β*≈0) and red (*α*≈0.999, *β*≈**−**1.86) stochastic series.

Figure (1) illustrates that changing the colour of AR(1) or 1/*f* stochastic series also leads to a change in the distribution shape. White series are normally distributed (fail to reject the null hypothesis of the Jarque-Bera test), while reddened AR(1) or 1/*f* series tend not to be. There is an increasing probability that blue AR(1) series fail the normality test. These results were driven by changes in the variance of the sample skewness and kurtosis statistics with colour (Fig. S1 in Supporting Information). There is also a decrease in the mean kurtosis associated with blue and reddened AR(1) and reddened 1/*f* models (Fig. S1). These qualitative results also held over longer (10^7^ steps) and shorter (100 steps) series.

As both AR(1) and 1/*f* methods generate non-normally distributed coloured series over finite time-scales, we used spectral mimicry [19] to generate ‘control’ coloured environmental series with a desired, normal frequency distribution. Briefly, spectral mimicry takes two input series of equal length, *X* and *Y*, and reorders one series (*Y*) to generate a third series (*Z*) that approximates the temporal characteristics (colour) of the other (*X*). For each replicate here, a traditional coloured series (*X*) was generated as described above, with an independent, random series (*Y*) drawn from a standard normal distribution (mean = 0, standard deviation = 1). Only random series, *Y*, that failed to reject the null hypothesis of a Jarque-Bera statistical test (data are normally distributed; significance level *a* = 0.05) were selected for further use. The elements of *X* were ranked in increasing value, with their *order statistics* recorded from the original series. Series *Z* is then generated from *Y* “by replacing each element of *Y* by the corresponding *order statistic* of *X*.” ([19] p. 433). This algorithm results in series *Z* having a spectral exponent similar to that of *X* within the limits examined here. In practice, series generated with spectral mimicry showed *β*<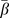 in very red environments, but otherwise, mimicry performed well across the time-scales examined here (Fig. S2).

Traditional series (*ε* = *X*) were generated with *T* = 10,000 steps for each desired value of 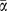 and 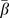, replicated 1000 times for each parameter value. Corresponding, normally distributed ‘control’ series (*ε = Z*) were generated for each replicate using spectral mimicry. All series were rescaled to zero mean and desired variance at the given time scale, following the methods set out in [14]. Three levels of environmental variance were examined in analyses of population fluctuations, *σ_ε_* ^2^ = 0.01, 0.1 and 0.5, to investigate any interaction between the size of environmental fluctuations and the non-linear deterministic population model (eqn. 2, below). Values of *σ_ε_* ^2^>0.5 were found to induce pseudo-extinctions in population simulations due to computational numerical precision limits.

In the Supporting Information, we illustrate the effects of controlling the distribution shape on the probability of generating series of extreme events (Fig. S3). Changes in the skewness and kurtosis with environmental colour have important consequences on the distribution shape even at shorter time-scales (*T* = 500). These analyses demonstrate that previous results based on traditionally generated AR(1) methods, showing changes in the probability of single or series of extreme events with colour [12] are artefacts of the changes in the shape of the frequency distribution. The same artefacts arise with 1/*f* methods. Single extreme events do not become less likely as the environment reddens (*α*>0, *β*<0) if coloured series are controlled to be normally distributed. Likewise, series of extreme events do not become less likely in red environments (Fig. S3).

### Population Fluctuations and Extinction Risk

Traditional and ‘control’ AR(1) or 1/*f* environmental series were generated, with colour parameters distributed over 31 evenly spaced values across the ranges 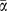 = [±0.999], 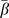 = [–2, 1] (AR[1] methods do not generate blue series with a sample spectral exponent *β*>1). These series were used to force a simple population growth function, the commonly used discrete-time theta-logistic (Ricker) function, which models population density (*N*) over consecutive time steps (*t*) as.
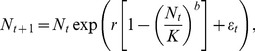
(2)where *r* is the intrinsic growth rate (*r* = 1.5), *K* is the carrying capacity (*K* = 100; results here are not qualitatively affected by the choice of this value when *K*>0) and *b* describes the shape of density dependence (*b* = 0.1). These parameter values result in undercompensatory (slow) population dynamics, allowing comparison with previous work (see also Fig. S4). The population response to environmental variability is given by *ε_t_*, modelled as either traditional AR(1) or 1/*f* processes, or through spectral mimicry based on random, Gaussian processes as described above.

Previous work has used *N_t_*_+1_ as the expected value of a Poisson random process to model demographic stochasticity and explicit extinction events, coupled with stochastic fluctuations entering through the carrying capacity, *K_t_* = *K*_0_+ *ε_t_* [8], [9], [11], [12]. However, results generated under those conditions are sensitive to certain model assumptions, e.g., the minimum value of *K_t_* [12], which requires limiting *K_t_*≥0 to avoid biologically unfeasible (complex valued) dynamics. This earlier work considered environmental fluctuations with very high variance, leading to high probabilities of negative *K_t_* values unless truncated. For example, the cumulative probability of *K_t_*≤5 is ∼0.07 in coloured series with *K*_0_ = 100, *σ_ε_* ^2^ = 4140 (values used in the above studies), assuming a normal frequency distribution. To avoid these issues, we considered the more general case where environmental variation affected *per capita* growth rate (*pgr*) additively and recorded the Coefficient of Variation of population fluctuations, *CV_N_* = *σ_N_*/*µ_N_*. While this did not generate explicit extinction events across the range of *σ_ε_* ^2^ values considered here, results based on *CV_N_* captured important features of those models based on traditional methods of generating environmental fluctuations that incorporated explicit extinctions. Therefore, *CV_N_* is used here as a proxy for extinction risk. While other methods exist for estimating population extinction risk (reviewed in [20]), we concentrate here on the relative size of population variability (*CV_N_*), as this is easily obtained from and commonly used in empirical time series analysis [21], but see [22] for an exception under complex (chaotic) deterministic dynamics.

Results for population fluctuations are presented below as a function of sample (output) autocorrelation coefficients (*α*) or spectral exponents (*β*) from each environmental time-series, rather than the desired (input) values (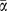, 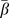). These results were presented by grouping sample *β* values in 25 evenly spaced bins between the limits *β* = [–2, 1]. Time-series based on values outside these limits were excluded from further analysis.

## Results

### Population Fluctuations and Extinction Risk

**Figure 2:**
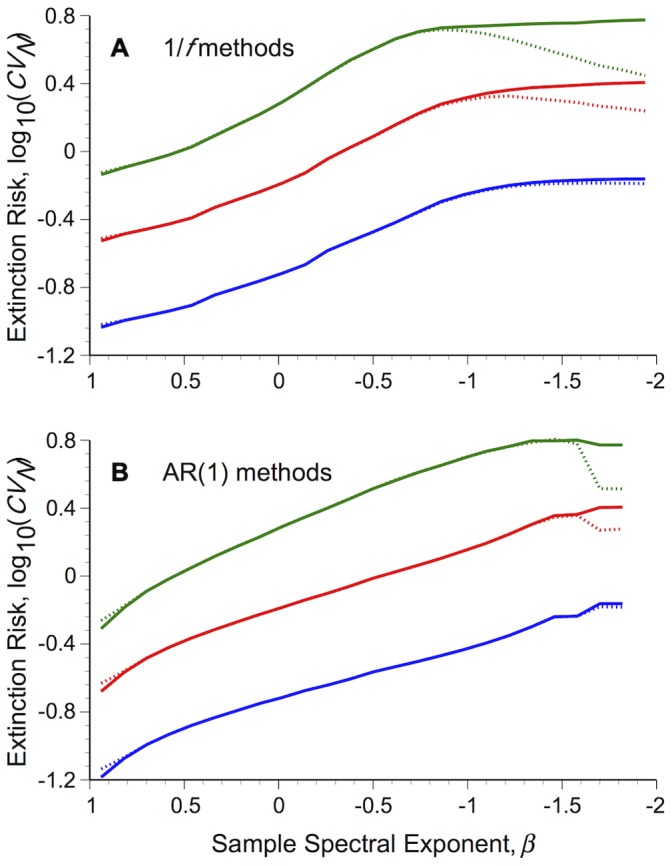
Population extinction risk varies with environmental colour, but changing environmental distribution shape confounds patterns. Undercompensating populations were forced by coloured environmental stochasticity modelled as either (A) 1/*f,* or (B) AR(1) processes. Dashed lines show the coefficient of variation (*CV_N_*) of population fluctuations based on environmental series generated with traditional methods, solid lines show results based on normally distributed series generated using spectral mimicry. Populations were iterated over 10,000 steps, forced with with *σ_ε_^2^*(*T*_10,000_) = 0.01 (blue lines), 0.1 (red lines) or 0.5 (green lines). Results show the median *CV_N_* value, based on sample environmental spectral exponents (*β*) binned into 25 evenly spaced groups between the limits [–2, 1], drawn from 1,000 replicates for each desired colour statistic, distributed between *α* = [–0.999, 0.999] and *β* = [–2, 1]. Population parameters: *r* = 1.5, *b* = 0.1, *K* = 100. Values along the *x*-axis have been reversed for easier comparison.

When the deterministic undercompensatory population model (eqn. 2) is forced by intermediate or strong environmental stochasticity [*σ_ε_* ^2^(*T*_10,000_)≥0.1] generated with traditional methods, environmental reddening initially leads to an increase, followed by a decrease in the size of population fluctuations (*CV_N_*) for both 1/*f* and AR(1) methods (Fig. 2). This corroborates previous work using 1/*f* models based on slightly different assumptions [8]. It also extends the parameter space examined for AR(1) models there and elsewhere [9], [11], [12], revealing a qualitatively similar decline in population variability for very red AR(1) environments, as found with 1/*f* methods. Population variability tends to show an asymptotic increase with reddening for weaker environmental stochasticity [*σ_ε_* ^2^(*T*_10,000_) = 0.01] in both AR(1) and 1/*f* models.

Populations forced by normally distributed, coloured environmental fluctuations did not show these strong declines in variability in redder environments (Fig. 2). There was no decline in red 1/*f* environments and a relatively small decline under very red AR(1) environments. Extinction risk was slightly overestimated in blue environments (*β* → 1) generated with traditional methods (Fig. 2).

**Figure 3:**
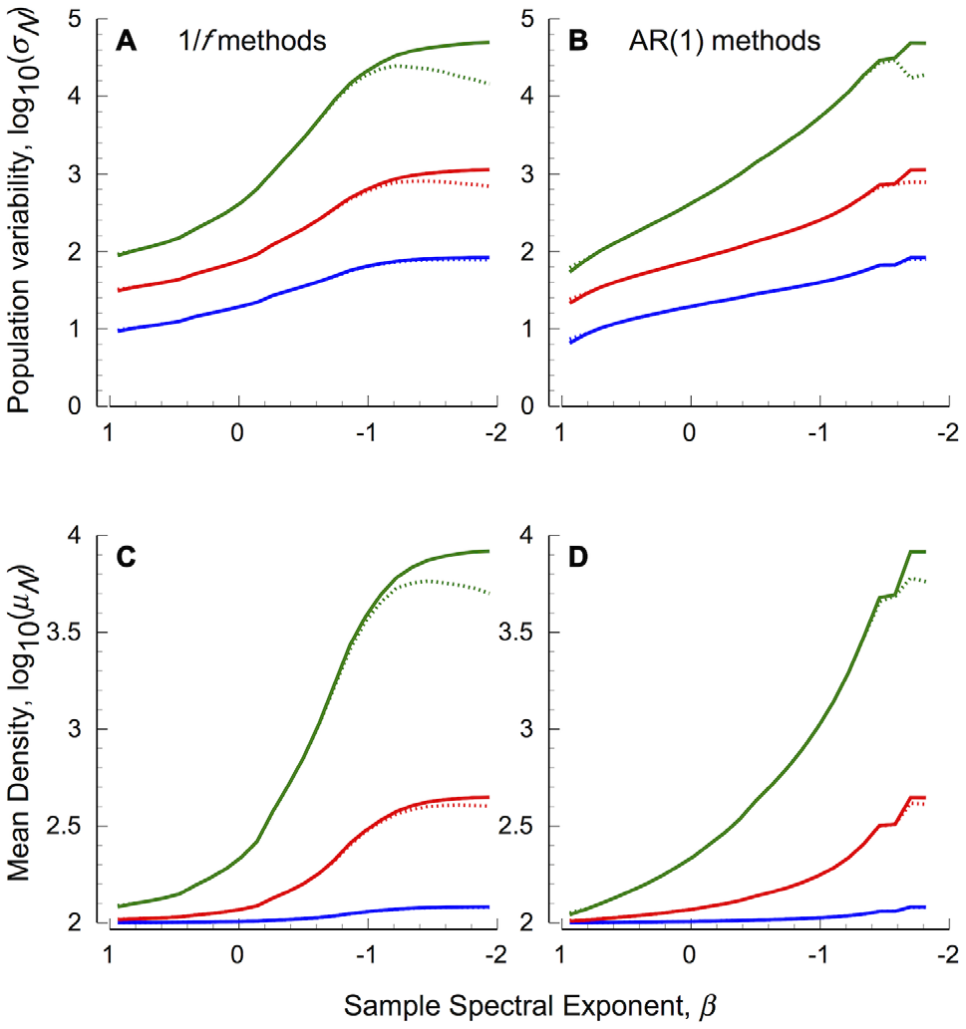
Statistical components of extinction risk for undercompensating populations forced by coloured environmental stochasticity. (A, B) show the standard deviation of population fluctuations, (C, D) show mean population densities. Left panels (A, C) show results based on 1/*f* stochastic processes, right panels (B, D) show AR(1) processes. Dashed lines show results based on environmental series generated with traditional methods, solid lines show results based on normally distributed series generated using spectral mimicry. Other details as in Figure 2.

Differences in *CV_N_* with changing environmental colour can be understood by examining the component population level statistics, *σ_N_ and µ_N_* (Fig. 3): *σ_N_* increases at a faster rate than *µ_N_*, resulting in the increase in extinction risk (*CV_N_*) from white to pink environments in all cases. The decrease in *CV_N_* under intermediate and strong (*σ_ε_* ^2^≥0.1) traditional pink to red environments occurs as *σ_N_* declines, despite the simultaneous decrease of mean population density in red 1/*f* environments and asymptote of *µ_N_* in red AR(1) environments. No such declines in *σ_N_* are present in pink to red environments controlled to have a normal distribution (Fig. 3).

**Figure 4:**
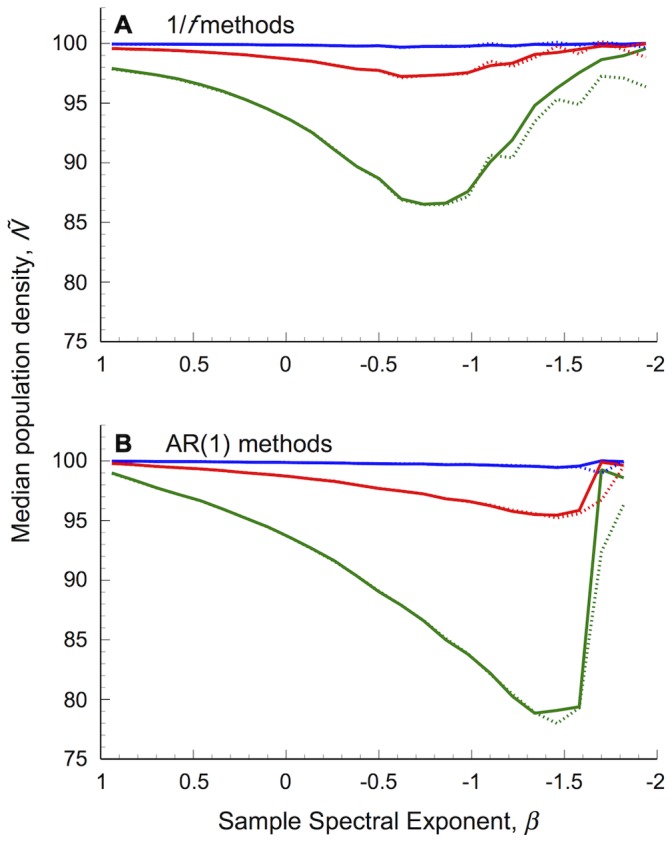
Median population density for undercompensating populations subjected to coloured environmental stochasticity. The environment was modelled as either an (A) 1/*f* or (B) AR(1) processes. Dashed lines show results based on environmental series generated with traditional methods, solid lines show results based on normally distributed series generated using spectral mimicry. Other details as in Figure 2.

While mean population density tends to increase with environmental reddening, median densities are generally below the carrying capacity (*K* = 100) when *σ_ε_* ^2^≥0.1 (Fig. 4), indicating a strong skew in population densities. Strongly concave density dependence (generating undercompensatory dynamics; see Fig. S4) means that *per-capita* growth rates decline rapidly from high densities (*N_t_*>*K*) but increase relatively slowly from very low densities (*N_t_*<*K*).

Therefore, traditionally generated coloured stochastic (environmental) series cause underestimation of extinction risk in reddened environments through changes in the distribution shape of the environmental signal, rather than colour *per se*, which lead to reduced population variability and increased skew in population fluctuations.

## Discussion

We have shown here that traditional AR(1) and 1/*f* methods for generating coloured stochastic (environmental) series also tend to produce coloured series that are not normally distributed over finite lengths (temporal or spatial scales), even though they do generate normally distributed white series. These changes in distribution shape confound the effect of colour on population dynamics in unexpected ways, leading to an underestimation of extinction risk in red (slowly changing) environments.

Using only normally distributed environmental series removes the confounding effect of two contrasting components of environmental variability present in very red environments generated with traditional methods: the patterns in the probability of single and series of poor conditions with environmental reddening ([12], Fig. S1), which lead to a decrease in extinction risk (reduced *CV_N_;* Fig. 2). This means that changes in the frequency distribution shape of coloured stochastic (environmental) processes with environmental reddening result in underestimates of extinction risk for undercompensating populations in red environments.

Cuddington and Yodzis [8] noted reductions in extinction risk (increased persistence time) in red and brown environments with traditional 1/*f* environmental models. Results presented here illustrate that (*i*) contrary to previous predictions ([8], [9], but see [12]), environmental stochasticity modelled with traditional AR(1) models can also generate reductions in extinction risk in very red environments (corresponding to *β*>1.5) and (*ii*) these reduced extinction risks are largely driven by changes in frequency distribution shape, rather than environmental reddening *per se*. The second result is confirmed by modelling normally distributed environmental fluctuations with the spectral mimicry method [19].

Changes in the frequency distribution shape of coloured stochastic variables risk violating one common assumption of the approach used to model the impact of environmental variation on population fluctuations: that stochasticity is normally distributed [10], [15]. Such changes could lead to a confounding effect that may require re-examination of previous simulation based results, including those that examine structured population models [6], [8]–[11], [23]–[29]. Alternative distribution types were not consistent across the range of environmental colours examined here, making general characterisation difficult. Roughgarden [13] pointed out that the choice of distribution for the random component *φ_t_* is arbitrary in the AR(1) generating method (eqn. 1). It remains crucial to consider whether it is a change in the colour *per se*, or a change in the distribution shape of the environmental noise that drives changes in population behaviours.

Pink noise has been suggested as a null model for the environmental variation forcing ecological dynamics [6], [30]. It is also worth considering what the null distribution should be. Results here show that when commonly used methods depart from a normal distribution, incorrect inferences can be drawn about extinction risks in reddened environments (Fig. 2). As yet, there is no consensus over what methods should be used to generate ‘true’ 1/*f* type processes [31], leaving open the question of what distribution shape should be expected under pink or red noise.

AR(1) and 1/*f* processes differ in their ‘memory’ properties when generating coloured noise, i.e., *α*, *β* ≠ 0 [32]: the memory of past conditions (autocovariance function) in 1/*f* processes tends to decay more slowly than AR(1) processes. Do these particular differences result in qualitatively different effects when filtered through density dependent [AR(1)] population dynamics? Results here suggest any qualitative differences are reduced, or disappear, when normally distributed fluctuations are filtered through undercompensating population dynamics (Fig. 2). Many of these issues only become apparent over longer time-scales than ecologists typically have available data. For example, running simulations over only 30 time-steps produces no discernible differences between *CV_N_* under traditional and spectral mimicry generating methods (results not shown). This does not imply that model results based on longer time-scales are not relevant, however. Empirical data limitations should not be confused with long-term natural population behaviours.

The power-law relationship between temporal lags and autocorrelation coefficients has been suggested as a proxy for the memory of a time series, another potential factor that can help explain persistence time and the pattern of time-series fluctuations [1], [30]. There are known problems with commonly used methods for estimating power-law exponents [33] and questions over the statistical robustness of reported power-laws [34]. Alternative approaches are unlikely to be useful in studies of explicit extinction events [33]. When extinctions occur over very short time scales (e.g., Cuddington and Yodzis [8] and Schwager et al. [12] both show extinction risk within 10 time steps of initiation increases with environmental reddening), there will be high uncertainty associated with the estimated autocorrelation or spectral coefficients and/or the ‘memory’ exponent [30]. Fractal estimates have been proposed as a robust method for characterising short, coloured time-series [30], [35], and may be worthy of consideration in studies that examine explicit extinction events (but see [36]), but strong, negative temporal trends are more likely to be behind the rapid (explicit) extinction events outlined in [8], [12].

Other model assumptions can also make comparison between studies awkward. For example, the form of the deterministic population model can have important effects, particularly under the influence of relatively strong environmental variability. The theta-Ricker model we used here has also been used in earlier studies on the impact of coloured noise [8], [9], [11]. Schwager et al. [12] examined a slightly different, non-linear population model, which can also show undercompensating dynamics [37]. While it is possible to choose parameter values that can make population behaviour close to the equilibrium similar for different models, the above studies have examined strong environmental fluctuations that move the population relatively far in phase space from the equilibrium point, where the functional forms can show important differences (Fig. S4).

The probability and frequency of catastrophic events is of great relevance to conservation biology. Catastrophic events can be thought of as representing the ‘tails’ of a distribution of population or environmental fluctuations [38], [39]. Predicted extinction risks for specific populations should be based on estimates of both the structure of the deterministic dynamics driving that population and the environmental covariates that are important for that population. We must collect further data on the colour and the distribution shape of the actual environmental covariates that drive population fluctuations as well as the functional form of population responses to environmental variation e.g., [40] before general conclusions can be drawn. The environmental time-series used here can also be thought of as the combined response of the population to various changes in the environment, and/or a particular biotic or abiotic variable, e.g., temperature or rainfall. Microcosm experiments have confirmed that environmental reddening does affect the size of population fluctuations [24], [41], [42], while analyses of natural time-series have shown that there is a wide range of spectral exponents in population data, with the majority lying between white and red [43]. Separating the intrinsic and extrinsic drivers behind the observed population fluctuations in time-series data remains a formidable challenge in population biology (but see [44], [45]).

The structure of stochastic (environmental) variation has implications across a variety of areas within population biology and beyond [1]–[4]. For example, the frequency distribution shape of spatio-temporal environmental structure should be carefully considered when trying to understand bet-hedging strategies in stochastic eco-evolutionary systems (e.g., [46]–[48]). The ability to anticipate future conditions changes with environmental colour, becoming less predictable as the environment becomes whiter (|*α*|, |*β*| → 0).

Further research is needed to fully understand the mechanisms driving extinctions in natural populations and the importance of coloured noise filtering through biological processes to drive population fluctuations. Results here show that previously reported patterns of extinction risk in density dependent population models can arise as an artefact of changes in the distribution shape of environmental fluctuations, rather than environmental colour. We can now interpret previous and future theoretical results in a new light, accounting for the effects of changes in the underlying distribution of environmental covariates as well as the environment’s temporal structure.

## Supporting Information

Figure S1 **Skewness and Kurtosis measures from (A, C) AR(1) and (B, D) 1/** ***f*** **coloured stochastic series (** ***T*** **= 10,000 steps; 1,000 replicates for each parameter value).** Reddened series (*α*>0, *β*<0) show an increased variance in both Skewness and Kurtosis values (blue line = mean), with a reduced mean Kurtosis for very red and blue AR(1) and pink to red 1/*f* models. AR(1) models also show increased variance for Kurtosis under blue noise (*α*<0).
(PDF)

Figure S2 **Comparing the expected (traditional method) and observed (spectral mimicry) colour statistics (top row: spectral exponents,** ***β*** **; bottom row: autocorrelation coefficients,** ***α*** **) in stochastic series generated using 1/** ***f*** **(left column) and AR(1) (right column) models.** Each black point represents the relationship between expected and observed colour statistic for a single replicate. The blue line shows the 1∶1 relationship.
(PDF)

Figure S3 **The probability of single and runs of** ***n*** **extreme values (** ***ε_t_*** **≤−2.5) occurring in AR(1) and 1/** ***f*** **coloured environmental series varies with environmental reddening (increasing** ***α*** **,** ***β*** **), for 21 values of** ***α*** **between the limits [0, 0.999] or** ***β*** **∼[0, 2].** (*a*, *b*) Probability of a single value *ε_t_* ≤ –2.5 in coloured series scaled to *σ_ε_* ^2^(*T*_∞_) = 1 (solid lines) or *σ_ε_* ^2^(*T*_500_) = 1 (dashed lines) in a 500 step sequence [1/*f* series scaled to *σ_ε_* ^2^(*T*_∞_) = 1 behave very differently, results not shown]. Panels (c–f) show the probability of finding *n* = (2, 3, 5 or 9) consecutive values of *ε_t_* ≤ –2.5 in a 500 step sequence scaled to *σ_ε_* ^2^(*T*_500_) = 1 using (c, d) traditional or (e, f) spectral mimicry methods.
(PDF)

Figure S4 **Per-capita growth rates for two different population models with the same carrying capacity (** ***K*** **= 100).** Based on (A) the original parameter values for the theta-Ricker model (blue line: *r* = 1.5, *b* = 0.1) or *MSS* model by Schwager et al. (2006; green line: *λ* = 4.5, *b* = 0.5) and (B) parameter values chosen to maintain identical behaviour around the equilibrium point for both models: theta-Ricker (*r* = 0.5, *b* = 0.3), *MSS* (*λ* = 2, *b* = 0.3). Scaling parameter values to give identical dynamics around the equilibrium does not ensure that dynamics will be similar elsewhere in the population phase space.
(PDF)

Text S1 (DOC)
