# Supplementary figures and images for: Confounding Environmental Colour and Distribution Shape Leads to Underestimation of Population Extinction Risk
Source: PLoS One. 2013 Feb 11;8(2):e55855. doi: 10.1371/journal.pone.0055855 (PMC3569452; doi:10.1371/journal.pone.0055855)

AR(1) model

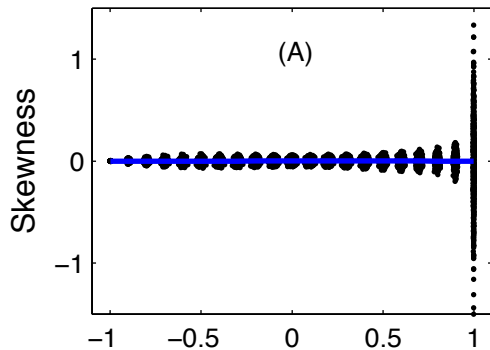 $1/f$  model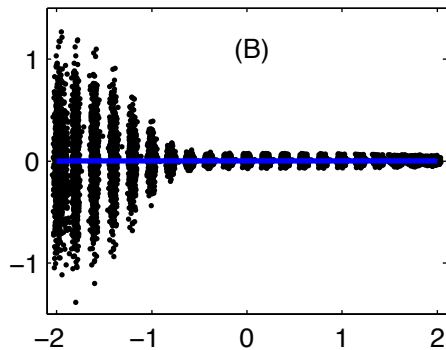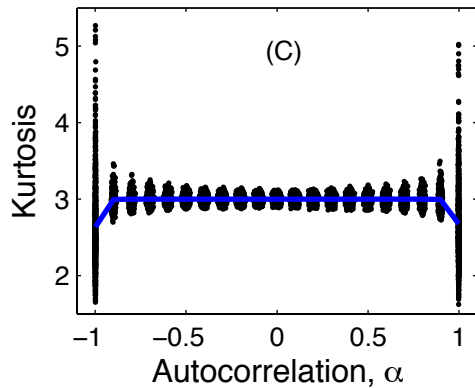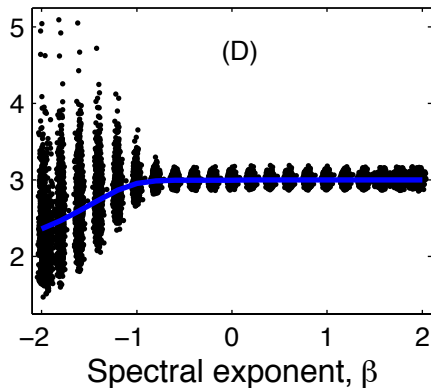

Supplement: Figure S1 — Skewness and Kurtosis measures from (A, C) AR(1) and (B, D) 1/ f coloured stochastic series ( T = 10,000 steps; 1,000 replicates for each parameter value). Reddened series (α>0, β<0) show an increased variance in both Skewness and Kurtosis values (blue line = mean), with a reduced mean Kurtosis for very red and blue AR(1) and pink to red 1/f models. AR(1) models also show increased variance for Kurtosis under blue noise (α<0). (PDF) [file pone.0055855.s001.pdf]

1/f model

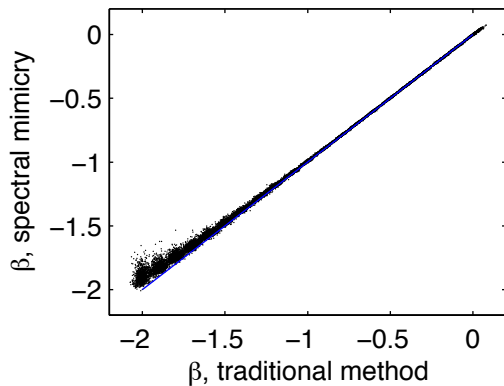

AR(1) model

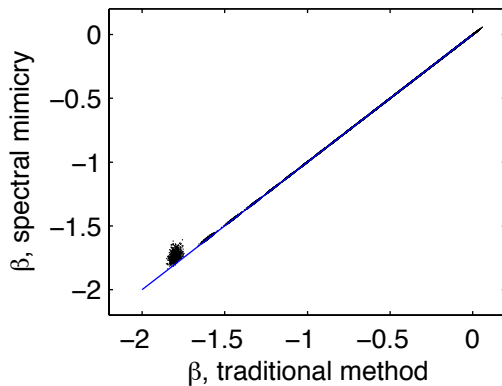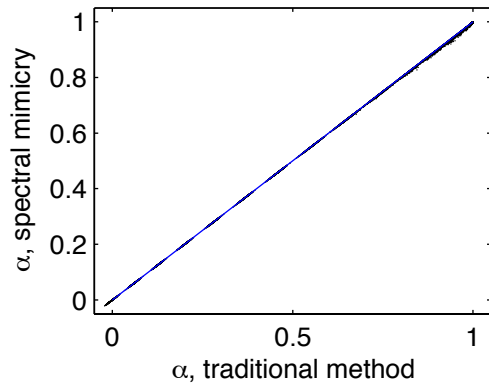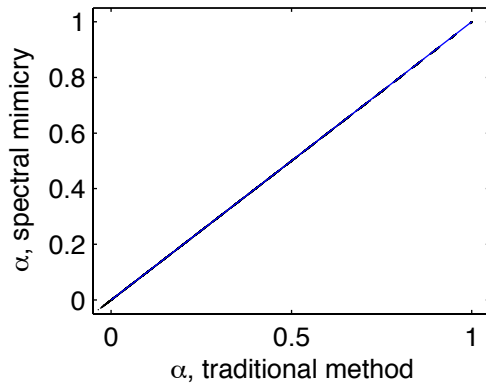

Supplement: Figure S2 — Comparing the expected (traditional method) and observed (spectral mimicry) colour statistics (top row: spectral exponents, β ; bottom row: autocorrelation coefficients, α ) in stochastic series generated using 1/ f (left column) and AR(1) (right column) models. Each black point represents the relationship between expected and observed colour statistic for a single replicate. The blue line shows the 1∶1 relationship. (PDF) [file pone.0055855.s002.pdf]

Traditional AR(1)

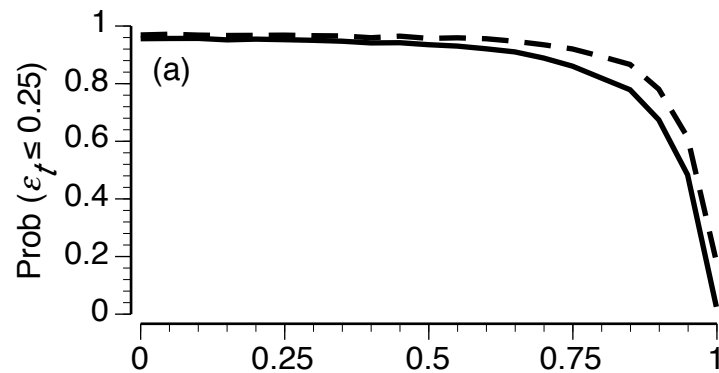Traditional  $1/f$ 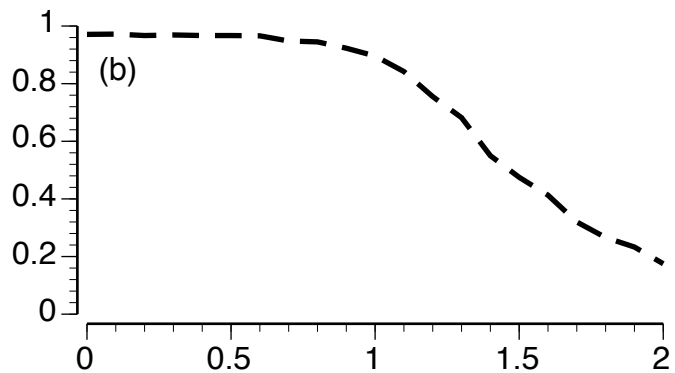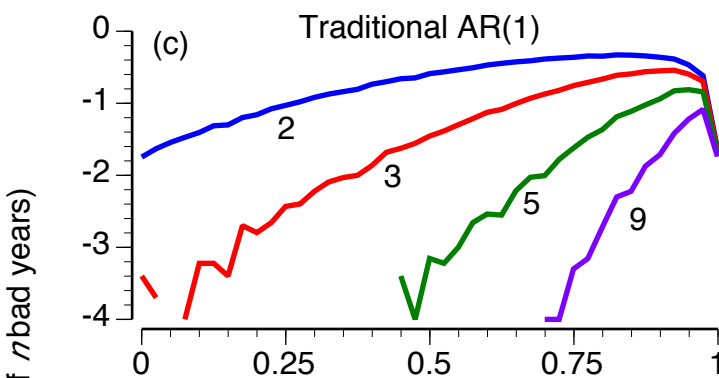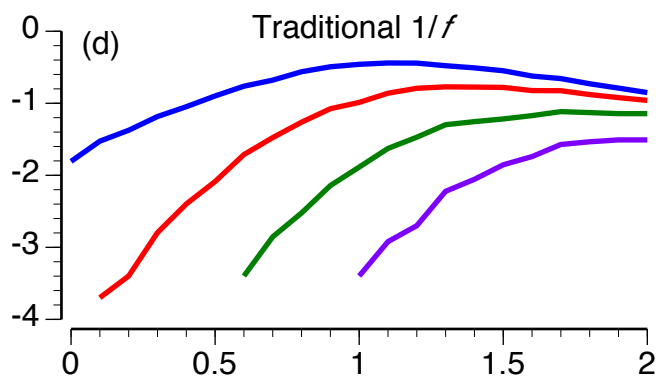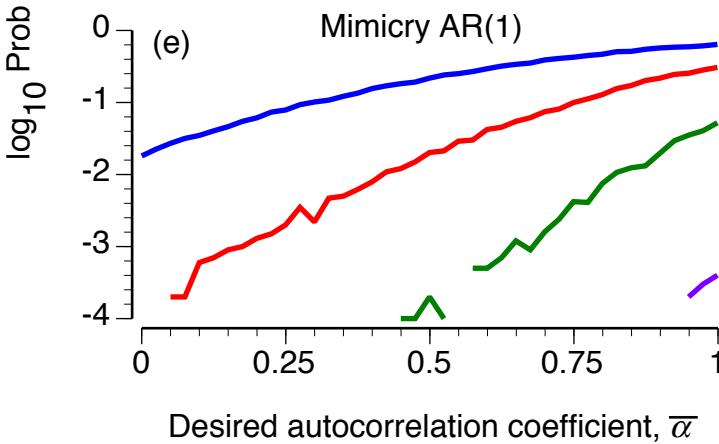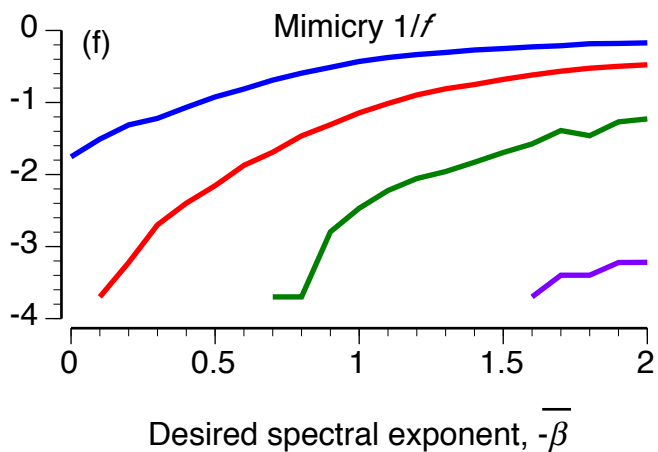

Supplement: Figure S3 — The probability of single and runs of n extreme values ( εt ≤−2.5) occurring in AR(1) and 1/ f coloured environmental series varies with environmental reddening (increasing α , β ), for 21 values of α between the limits [0, 0.999] or β ∼[0, 2]. (a, b) Probability of a single value εt ≤ –2.5 in coloured series scaled to σε 2(T ∞) = 1 (solid lines) or σε 2(T 500) = 1 (dashed lines) in a 500 step sequence [1/f series scaled to σε 2(T ∞) = 1 behave very differently, results not shown]. Panels (c–f) show the probability of finding n = (2, 3, 5 or 9) consecutive values of εt ≤ –2.5 in a 500 step sequence scaled to σε 2(T 500) = 1 using (c, d) traditional or (e, f) spectral mimicry methods. (PDF) [file pone.0055855.s003.pdf]

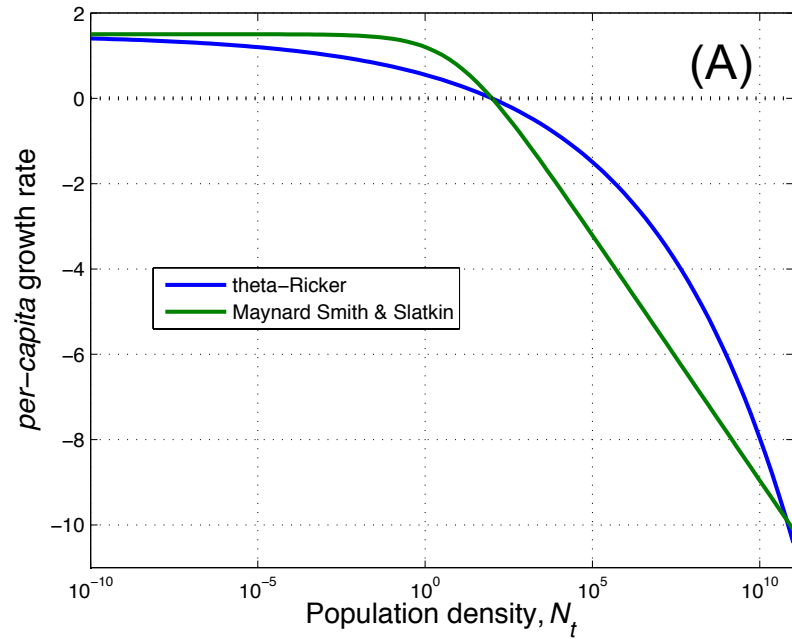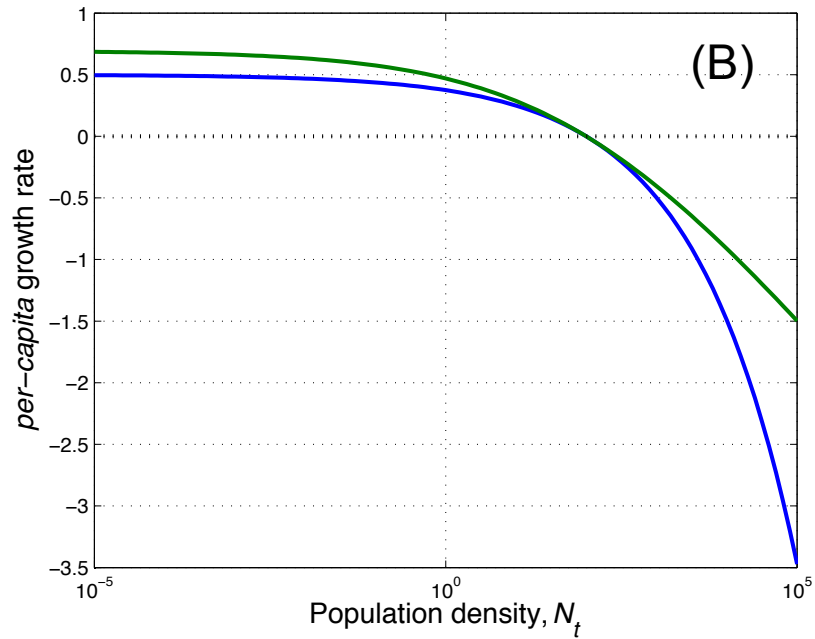

Supplement: Figure S4 — Per-capita growth rates for two different population models with the same carrying capacity ( K = 100). Based on (A) the original parameter values for the theta-Ricker model (blue line: r = 1.5, b = 0.1) or MSS model by Schwager et al. (2006; green line: λ = 4.5, b = 0.5) and (B) parameter values chosen to maintain identical behaviour around the equilibrium point for both models: theta-Ricker (r = 0.5, b = 0.3), MSS (λ = 2, b = 0.3). Scaling parameter values to give identical dynamics around the equilibrium does not ensure that dynamics will be similar elsewhere in the population phase space. (PDF) [file pone.0055855.s004.pdf]
