# Supplementary material for: Confounding Environmental Colour and Distribution Shape Leads to Underestimation of Population Extinction Risk
Source: PLoS One. 2013 Feb 11;8(2):e55855. doi: 10.1371/journal.pone.0055855 (PMC3569452; doi:10.1371/journal.pone.0055855)
Supplement: Text S1 — (DOC) [file pone.0055855.s005.doc]

Supporting Information

Mike S. Fowler & Lasse Ruokolainen

Confounding environmental colour and distribution shape leads to underestimation of population extinction risk.

First we present the sample Skewness and Kurtosis statistics for coloured stochastic processes generated with traditional AR(1) and sinusoidal (1/f) methods (Fig. S1). These are based on 1,000 replicates of 10,000 step series for each parameter value examined (distributed in 21 evenly spaced steps between the limits α ~ [± 0.999]; β ~ [±2]). Red environments are associated with an increasing variance in both Skewness and Kurtosis and a decrease in the mean Kurtosis for both AR(1) and 1/f methods (Fig. S1). AR(1) methods also show an increased variance and decrease mean Kurtosis in blue environments.

Comparing colour characteristics of traditional and spectral mimicry methods

Here we compare the expected (input) and observed (sample) autocorrelation coefficients (α) and spectral exponents (β) for time series generated with traditional or spectral mimicry (Cohen et al. 1999) methods (Fig. S2). Spectral mimicry does a very good job of generating series with similar sample colour statistics (α and β) for the whole range of AR(1) values and for white to pink noise generated with 1/f methods over time-scales investigated here (T = 10,000). However, β tends to be underestimated in very red environments. Spectral mimicry was not capable of generating brown or black series (β > 2) over this time-scale (results not shown), even with input (sample) β values > 2.

The probability of single and series of extreme events

Previous work has shown that the probability of a single extreme event occurring in a coloured environmental series decreases with the autocorrelation coefficient, α, in AR(1) models (Schwager, Johst, & Jeltsch 2006). The variance of AR(1) processes is known to change predictably at both finite and infinite time-scales (Roughgarden 1975; Heino, Ripa, & Kaitala 2000) with similar results for 1/f processes (Halley & Inchausti 2004).

Schwager et al. (2006) also reported an initial increase in the probability of n > 1 consecutive events occurring beyond some critical threshold (e.g., μ_ε_ ± 2.5σ_ε_^2^) from white to pink environments, followed by a decrease in the likelihood of such runs in very red environments. This result also has the potential to be biased by two separate mechanisms: (1) changes in the variance of stochastic processes with colour over finite time-scales; and (2) changes in the distribution shape (skewness and kurtosis) of ε with colour over finite time-scales.

The influence these mechanisms have on the probability of single or a series of extreme events occurring can be investigated by comparing traditional AR(1) environmental series with variance scaled at infinite time-scales [eqn. 1; σ_ε_^2^(T_∞_) = 1], with those where variance was scaled at finite time-scales [σ_ε_^2^(T_500_) = 1]. Coloured series generated with spectral mimicry were also used to test the effect of changes in distribution shape with colour on results when n > 1. These series were scaled to σ_ε_^2^(T_500_). Following Schwager et al. (2006), AR(1) series were iterated over 1,500 steps, with the first 1,000 steps discarded. The remaining 500 steps were either tested for the presence of n = (1, 2, 3, 5, 9) consecutive values ε_t_ ≤ –2.5 without further scaling, or rescaled to mean 0 and the variance σ_ε_^2^(T_500_) = 1. Results here were based on 10,000 replicated series for each value of α. We also show results based on 1/f methods as outlined above, rescaled to σ_ε_^2^(T_500_) = 1 here for completeness.

Scaling AR(1) environmental variance to σ_ε_^2^(T_500_) = 1 does not change the qualitative reduction in the probability of single extreme events (ε_t_ ≤ –2.5) that occurs with environmental reddening when σ_ε_^2^(T_∞_) = 1. However, the effect is slightly reduced when σ_ε_^2^(T_500_) = 1 (Fig. S3a). The general decline in the probability of single extreme events is also apparent with 1/f methods when σ_ε_^2^(T_500_) = 1 (Fig S3b). Changes in the skewness and kurtosis therefore have important impacts on the distribution shape and probability of single extreme events even at relatively short time-scales.

Environmental reddening leads to an initial increase, then decrease in the probability of finding a run of extreme values using traditional methods of generating coloured AR(1) or 1/f series when scaled at σ_ε_^2^(T_500_) = 1 (Fig. S3c,d), confirming previous results based only on AR(1) models (Schwager et al. 2006). Controlling the distribution shape of the environmental series through spectral mimicry reveals that the decline in the probability of finding long runs of values in very red environments is again an artefact of changing frequency distribution shapes under traditional methods (Fig. S3e,f). Scaling the variance of ε at infinite time-scales [σ_ε_^2^(T_∞_) = 1] does not qualitatively alter these results.

Comparing different population growth functions

Schwager et al. (2006) studied a slightly different model formulation than that considered in the main text, based on the Maynard Smith & Slatkin (1973) [MSS] model:

|  | . | (S1) |
| --- | --- | --- |

The deterministic functional form of MSS differs from the theta-Ricker function used to investigate population dynamics in the main text (eqn. 2, main text) and elsewhere (e.g., Petchey, Gonzalez, & Wilson 1997; Cuddington & Yodzis 1999; Heino et al. 2000), given the parameter values used by Schwager et al. (2006): λ = 4.5, K = 100, b ≥ 0.5. These differences can be illustrated by examining the per-capita growth rates (pgr) of the two functions (Fig. S4). The MSS model shows higher rates of increase when the population density is below equilibrium (N < K) and more rapid declines when N > K – i.e., MSS with these parameters is less undercompensatory: populations grow (or decline) faster than under the theta-Ricker model across most of the population phase space.

One approach to facilitate comparison between these models would be to select relevant parameter values that could provide similar dynamics around the equilibrium (N* = K) for both models. This requires changing the parameter values used in either model, to provide equality when solving for the derivatives of the two pgr's:

|  | , | (S2) |
| --- | --- | --- |
|  | , | (S3) |

where f_1_ and f_2_ are the pgr functions, with their derivatives evaluated at equilibrium (N*) for the MSS (eqns. S1, S2) and theta-Ricker (eqns. 2, S3) functions respectively, giving λ = 1/(1 – r). This sets a limit of r < 1 to ensure biologically meaningful dynamics in the MSS function. It is possible to solve the equality for eqns. (S2 & S3) based on the parameter combination used in the main text (1 – br = 0.85) by setting e.g., r = 0.5 and b = 0.3, giving 1 – b(λ – 1)/λ = 1 – br = 0.85. Therefore, λ = 2 gives identical dynamics in the region of the interior equilibrium point. However, this does not necessarily generate similar dynamics at population densities further from the equilibrium (Fig S4B). Here, MSS tends to increase faster from low densities and decrease slower from high densities than theta-Ricker dynamics. Therefore, these different models tend to generate somewhat different dynamics over a wide range of population densities, i.e., when forced by strong environmental variation.

The above discussion assumes parameter b can be considered to have an equal effect in the two population models, which (while not strictly true) makes a comparison more straightforward. Choosing independent parameters for each model (b_1_ and b_2_) and setting λ = r results in similar outcomes.

LITERATURE CITED :

Cohen, J.E., Newman, C.M., Cohen, A.E., Petchey, O.L. & Gonzalez, A. (1999) Spectral mimicry: A method of synthesizing matching time series with different Fourier spectra. Circuits Systems and Signal Processing, 18, 431–442.

Cuddington, K.M. & Yodzis, P. (1999) Black noise and population persistence. Proceedings of the Royal Society Series B–Biological Sciences, 266, 969–973.

Halley, J.M. & Inchausti, P. (2004) The increasing importance of 1/f-noises as models of ecological variability. Fluctuation and Noise Letters, 4, R1–R26.

Heino, M., Ripa, J. & Kaitala, V. (2000) Extinction risk under coloured environmental noise. Ecography, 23, 177–184.

Maynard-Smith, J. & Slatkin, M. (1973) The stability of predator-prey systems. Ecology, 54, 384–391.

Petchey, O.L., Gonzalez, A. & Wilson, H.B. (1997) Effects on population persistence: the interaction between environmental noise colout, intraspecific competition and space. Proceedings of the Royal Society of London Series B–Biological Sciences, 264, 1841–1847.

Roughgarden, J. (1975) A Simple Model for Population Dynamics in Stochastic Environments. American Naturalist, 109, 713–736.

Schwager, M., Johst, K. & Jeltsch, F. (2006) Does red noise increase or decrease extinction risk? Single extreme events versus series of unfavourable conditions. American Naturalist, 167, 879–888.
